# Response to commentary

**DOI:** 10.1186/s40560-021-00572-6

**Published:** 2021-09-15

**Authors:** Yasutaka Hirasawa, Taka-aki Nakada, Jiro Terada

**Affiliations:** 1 Department of Respirology, Graduate School of Medicine, Chiba University, 1-8-1 Inohana, Chuo-ku, Chiba, 260-8670 Japan; 2 Department of Emergency and Critical Care Medicine, Graduate School of Medicine, Chiba University, 1-8-1 Inohana, Chuo-ku, Chiba, 260-8670 Japan

## Abstract

This is a response to the issues raised in the commentary by Dr. Yifu Si et al.

## Response

We would like to thank Editor-in-Chief for the opportunity to respond to the issues raised in the commentary by Dr. Yifu Si et al. We also greatly appreciate their interest in our paper.

Following are the two concerns raised by them:

First, acute respiratory failure (ARF) is heterogenous and the predictive ability of lymphocytes in bronchoalveolar lavage fluid (BALF) can be different in each disease.

The etiology of ARF varies and is complicated; hence, comprehensive diagnostic investigation including BALF is required. We agree that it is important to conduct further study in order to confirm our result with an adequate number of subjects. However, in critically ill patients with ARF, confirming a differential diagnosis in sufficient numbers is challenging. Therefore, our findings may include important aspects present in the real-world clinical settings.

Second, Dr. Yifu Si et al. suggest the possibility that decreased mortality in interstitial lung disease (ILD) patients could be biased in our study; the difference between our results and a report of a larger multicenter observational study with four different cohorts indicates that interstitial lung abnormalities (ILA) are associated with a higher risk of all-cause mortality [1]. In this point, we partly agree with the concerns of Dr. Yifu Si et al. that our study may be biased due to its small sample size.

However, ILA, defined as the incidental computed tomography (CT) findings, is a larger and more biased concept than ILD, which is detailed in our study [2]. Indeed, in ILA, the usual interstitial pneumonia (UIP) pattern obtained using chest CT imaging significantly associated with increased mortality [3]. Idiopathic pulmonary fibrosis (IPF) is one of the major ILDs with UIP pattern that is known to have poor prognosis. In our study, acute exacerbation of IPF has been diagnosed using the definition in international working group report, which was not necessary BALF since 2016 [4]. The increased risk of mortality with ILA may in part be related to the poor prognosis of IPF. Because of this, the comparison between ILA and ILD in the context of our study is problematic as the mortality rates vary considerably.

## Data Availability

Not applicable.

## Abbreviations

ARF: Acute respiratory failure
BALF: Bronchoalveolar lavage fluid
CT: Computed tomography
ILA: Interstitial lung abnormalities
ILD: Interstitial lung disease
IPF: Idiopathic pulmonary fibrosis
UIP: Usual interstitial pneumonia
